# Water from the Rock

**Published:** 1881-09

**Authors:** 


### WATER FROM THE ROCK.

We had a paragraph last week alluding to the benefits of pure water, and the ease with which it can be obtained in many cases. Tunneling into the hillsides was mentioned as one method which has yielded satisfactory results. We find a record of such experience in the Lakeport *Bee-Democrat:* It is stated that generally an abundance of water can be obtained by tunneling horizontally into a hillside for a distance varying from 50 to 100 ft. A point should be selected some distance above the residence or place where water is desired, and with a comparatively light expense the water can be conveyed to any part of the premises in unceasing abundance. Irrigation is made possible and easy by this same process, and, in fact, its usefulness cannot be estimated. Henry Alter, of Paradise Valley, has given this subject practical proof, also D. M. Hanson, of East Lake, and the result is satisfactory to the last degree. The cost is no greater than digging a well; that is, for the same distance, and then, when water is found, the labor and expense of pumping is wholly avoided. Frequently water will be found but a few feet below the surface in digging a well, but even then the water is likely to be of an inferior quality. Not so with the tunnel; water secured by that means is invariably of the best.—*Pacific Rural Press.*

---

MILK.—This article contains all of the elements needed to sustain life in the young, and is one of the simplest in use—wholesome, if it is pure. Its purity depends on the health of the producer, and that largely on the quality of the food eaten. If at first pure it may become otherwise by carelessness. To keep it in tins made of lead and arsenic, if it becomes sour, especially, is to endanger the life of the family. If uncovered or in any way exposed to fresh paint, or to filth or poison in any form, it soon becomes affected by absorption, since water and all liquids containing it are grand purifiers of the air, these impurities being retained in the liquids. Never keep milk in a newly-painted pantry—only water, and that to be thrown out.—*Dr. J. H. Hanaford.*

---

THE only son of Wild Bill is reported to be in Deadwood. That can't be, as he was in Boston, Chicago, Denver and Galveston yesterday, while seventeen of him was hanging about Leadville saloons. The man at Deadwood is evidently an impostor.—*Boston Post.*

